# Guidelines for Reporting Oral Epidemiologic Studies to Inform Burden Estimation (GROESBE)

**DOI:** 10.1177/00220345241293410

**Published:** 2024-12-05

**Authors:** E. Bernabé, C.C. Salomon-Ibarra, W. Marcenes

**Affiliations:** 1 Institute of Dentistry, Queen Mary University of London, London, UK; 2 Affordable Health Initiative, London, UK

**Keywords:** dental caries, periodontal diseases, tooth loss, mouth, prevalence, incidence

## Abstract

High-quality prevalence and incidence studies of oral conditions are essential for estimation of disease burden and comparison of estimates among countries and over time, as well as for priority setting, resource allocation, and planning public health action. Existing systematic reviews of the epidemiology of untreated dental caries, severe periodontitis, and edentulism, carried out for the Global Burden of Disease study, showed inadequate and incomplete reporting of the measurement of oral conditions as well as a lack of consistency and comparability with other health conditions. These issues are more accentuated in studies from low- and middle-income countries. Studies must meet the highest standards so that these efforts do not waste resources. This report extends the Strengthening the Reporting of Observational Studies in Epidemiology (STROBE) guidelines to improve and standardize the reporting of descriptive oral epidemiologic studies of common oral conditions. The aim of the Guidelines for Reporting Oral Epidemiologic Studies to Inform Burden Estimation (GROESBE) is to promote transparency, clarity, and comparability of scientific reporting, specifically for prevalence and incidence studies of untreated caries, severe periodontitis, and edentulism. The GROESBE guidelines and checklist were developed following a structured and formal consultation process with a geographically diverse group of 23 individuals involved in the conduct and analysis of oral epidemiologic studies. GROESBE focuses on elements that are not included in STROBE, adding 14 specific recommendations to existing guidelines. They will facilitate reliable comparison of emerging prevalence and incidence data on untreated caries, severe periodontitis, and edentulism across settings worldwide and the synthesis of robust evidence to inform estimation of disease burden in future iterations of the Global Burden of Disease study.

## Introduction

The accurate estimation and description of common oral conditions constitute an essential responsibility of the oral health community (Hugo et al. 2021; Schmalz et al. 2022). High-quality prevalence and incidence studies of oral conditions are essential for estimation of disease burden, comparison among countries and over time, priority setting, resource allocation, and health planning (Bernabe et al. 2020). Our systematic reviews for the Global Burden of Disease (GBD) study have highlighted the incorrect and incomplete reporting in the measurement of oral conditions among published studies (Kassebaum et al. 2014a, 2014b, 2015). Although studies from high-income countries and low- and middle-income countries (LMICs) can be of poor quality—due to poor reporting, poor methodology, or both—quality matters the most for LMICs because the number of descriptive studies in LMICs is sparse. Furthermore, collecting high-quality data on oral conditions from LMICs is increasingly important because these populations are likely to be the ones where the greatest future need for health care will be required by the middle of this century (Bernabe et al. 2020).

While there is an abundance of research protocols to guide researchers, there is not a guideline for reporting oral epidemiologic studies to inform burden estimation. The Strengthening the Reporting of Observational Studies in Epidemiology (STROBE) guidelines (Vandenbroucke et al. 2007; von Elm et al. 2007) are widely used but were devised for reporting analytic studies, not descriptive ones. Furthermore, they lack details on how to measure and report common oral conditions. The World Health Organization (WHO; 2013) manual for oral health surveys focuses on planning and implementation, with a brief list of standard descriptive tables listed in Annex 9. Standards for reporting periodontitis prevalence and severity in surveys were proposed by the Joint EU/USA Periodontal Epidemiology Working Group in the last decade (Holtfreter et al. 2015). With reporting standards, there is a need to delineate ideal and alternate reporting metrics that everybody—not only dentists and dental researchers—can understand, readily put into practice, and describe to others (Hugo et al. 2021).

This report extends the STROBE guidelines to improve and standardize the reporting of descriptive oral epidemiologic studies of common oral conditions. The aim of the Guidelines for Reporting Oral Epidemiologic Studies to Inform Burden Estimation (GROESBE) is to promote transparency, clarity, and comparability of scientific reporting, specifically for person-level prevalence and incidence studies of untreated caries, severe periodontitis, and edentulism.

## Methods for Developing GROESBE

The guideline was developed following recommended methods (Moher et al. 2010; Spranger et al. 2022). The protocol was approved by Queen Mary University of London’s Research Ethics Committee (QMERC23.155) and registered prospectively in https://www.equator-network.org/. Full details of the methodology are presented in Appendix 1, which included a systematic search of standards for reporting descriptive studies and a Delphi panel with 23 individuals experienced in conducting or analyzing oral health surveys or using survey findings for research and/or policy.

## GROESBE Checklist

GROESBE was developed as an extension to the STROBE statement and follows the same structure (Table). When no specific GROESBE item is listed in the Table, this means that the original STROBE item alone suffices. The GROESBE checklist provides additional details for 10 of the 22 items on the STROBE checklist, including a suggested flowchart of participation. Here, we describe the additional recommendations for GROESBE that are not already outlined in detail in STROBE or other extensions. Examples for each GROESBE item are presented in Appendix 2. Editable versions of the Table and the participation flowchart are provided in Appendixes 3 and 4, respectively.

**Table.** STROBE Checklist and Additional GROESBE Items.

Item	Item No.	STROBE Recommendations	GROESBE Items
**Title and abstract**	1	(*a*) Indicate the study’s design with a commonly used term in the title or the abstract.	
		(*b*) Provide in the abstract an informative and balanced summary of what was done and what was found.	
**Introduction**			
Background/rationale	2	Explain the scientific background and rationale for the investigation being reported.	
Objectives	3	State specific objectives, including any prespecified hypotheses.	
**Methods**			
Study design	4	Present key elements of study design early in the paper.	Provide a full description of the study design.
Setting	5	Describe the setting, locations, and relevant dates, including periods of recruitment, exposure, follow-up, and data collection.	Describe the setting, locations, and dates of data collection.
Participants	6	(*a*) *Cohort study*—Give the eligibility criteria, and the sources and methods of selection of participants. Describe methods of follow-up. *Case-control study*—Give the eligibility criteria, and the sources and methods of case ascertainment and control selection. Give the rationale for the choice of cases and controls. *Cross-sectional study*—Give the eligibility criteria, and the sources and methods of selection of participants.	Describe the source population, eligibility criteria, and methods of selection of participants. For incidence studies, describe methods of follow-up.
		(*b*) *Cohort study*—For matched studies, give matching criteria and number of exposed and unexposed *Case-control study*—For matched studies, give matching criteria and the number of controls per case	
Variables	7	Clearly define all outcomes, exposures, predictors, potential confounders, and effect modifiers. Give diagnostic criteria, if applicable.	Outcomes—Describe the diagnostic criteria for each oral condition.
			7a. Describe the diagnostic criteria and case definition for untreated dental caries.
			7b. Describe the diagnostic criteria and case definition for severe periodontitis.
			7c. Describe the diagnostic criteria and case definition for edentulism.
Data sources/measurement	8	For each variable of interest, give sources of data and details of methods of assessment (measurement). Describe comparability of assessment methods if there is more than one group.	Give sources of data and details of methods of assessment (examination protocol, reliability assessment, and summary indices) for each oral condition.
Bias	9	Describe any efforts to address potential sources of bias.	
Study size	10	Explain how the study size was arrived at.	Explain how the sample size was determined.
Quantitative variables	11	Explain how quantitative variables were handled in the analyses. If applicable, describe which groupings were chosen and why.	
Statistical methods	12	(*a*) Describe all statistical methods, including those used to control for confounding.	12a. Describe how prevalence and incidence were estimated.
		(*b*) Describe any methods used to examine subgroups and interactions.	
		(*c*) Explain how missing data were addressed.	12c. Clearly describe how missing data were handled.
		(*d*) Cohort study—If applicable, explain how loss to follow-up was addressed. Case-control study—If applicable, explain how matching of cases and controls was addressed. Cross-sectional study—If applicable, describe analytical methods taking account of sampling strategy.	
		(*e*) Describe any sensitivity analyses.	
**Results**			
Participants	13	(*a*) Report numbers of individuals at each stage of study—eg numbers potentially eligible, examined for eligibility, confirmed eligible, included in the study, completing follow-up, and analysed.	Report the number of participants at each stage of the study and reasons for nonparticipation at each stage.
		(*b*) Give reasons for non-participation at each stage.	
		(*c*) Consider use of a flow diagram.	
Descriptive data	14	(*a*) Give characteristics of study participants (eg demographic, clinical, social) and information on exposures and potential confounders.	For incidence studies, report details of follow-up time.
		(*b*) Indicate number of participants with missing data for each variable of interest.	
		(*c*) *Cohort study*—Summarise follow-up time (eg, average and total amount).	
Outcome data	15	*Cohort study*—Report numbers of outcome events or summary measures over time.	Report the number of existing and/or new cases of the condition.
		*Case-control study*—Report numbers in each exposure category, or summary measures of exposure.	
		*Cross-sectional study*—Report numbers of outcome events or summary measures.	
Main results	16	(*a*) Give unadjusted estimates and, if applicable, confounder-adjusted estimates and their precision (eg, 95% confidence interval). Make clear which confounders were adjusted for and why they were included.	
		(*b*) Report category boundaries when continuous variables were categorized.	
		(*c*) If relevant, consider translating estimates of relative risk into absolute risk for a meaningful time period.	
Other analysis	17	Report other analyses done—e.g. analyses of subgroups and interactions, and sensitivity analyses.	
**Discussion**			
Key results	18	Summarise key results with reference to study objectives.	
Limitations	19	Discuss limitations of the study, taking into account sources of potential bias or imprecision. Discuss both direction and magnitude of any potential bias.	
Interpretation	20	Give a cautious overall interpretation of results considering objectives, limitations, multiplicity of analyses, results from similar studies, and other relevant evidence.	
Generalisability	21	Discuss the generalisability (external validity) of the study results.	
**Other information**			
Funding	22	Give the source of funding and the role of the funders for the present study and, if applicable, for the original study on which the present article is based.	

GROESBE, Guidelines for Reporting Oral Epidemiologic Studies to Inform Burden Estimation; STROBE, Strengthening the Reporting of Observational Studies in Epidemiology.

### GROESBE Item 4

#### Methods: Study Design—Provide a Full Description of the Study Design

Authors should report the study design so that it is clear whether data were collected at 1 point in time (cross-sectional study reporting prevalence), multiple points in time for different participants (repeated cross-sectional study reporting prevalence trends), or multiple points in time for the same participants (cohort study reporting incidence).

### GROESBE Item 5

#### Methods: Setting—Describe the Setting, Locations, and Dates of Data Collection

Authors should provide information on the setting (e.g., recruitment sites such as communities, schools, primary care practices, hospitals, care homes) and location (e.g., geographic area) where the study took place to assess the context and generalizability of the results. Authors should give an indication of the geographic coverage of the study, reporting whether it was local (i.e., single community), regional, or national. Any claims regarding representativeness of the study sample should be explained and well founded. Authors should also state the years when data collection took place.

### GROESBE Item 6

#### Methods: Participants—Describe the Source Population, Eligibility Criteria, and Methods of Selection of Participants. For Incidence Studies, Describe Methods of Follow-up

A description should be presented of the population from which participants were selected (i.e., general population, noninstitutionalized population, schoolchildren, working-age adults) and any eligibility criteria (i.e., inclusion and exclusion criteria). Eligibility criteria should be justified, as they could limit the representativeness of the sample (Locker 2000).

Both the method of recruitment (e.g., email/postal invitation, existing records, referrals, self-selection through advertisements) and sampling procedure should be reported. Any methods used to increase participation and retention of participants should also be reported. It must be stated whether a probabilistic sample was used (e.g., participants recruited by simple random, systematic, cluster, or stratified sampling or a combination of those as in a multistage sample) or a nonprobabilistic sample (e.g., convenience, WHO pathfinder survey).

For incidence studies, authors should state the length of follow-up. It is recommended to give details on how participants were recontacted (e.g., electronic methods, nonelectronic methods, record linkage) and whether similar procedures were used for all participants.

### GROESBE Item 7

#### Methods: Outcomes—Describe the Diagnostic Criteria for Each Oral Condition

Authors should provide a definition of each oral condition being assessed (see items 7a to 7c for details on each oral condition). This should include a concise description of the detection criteria used, as supported by a reference for their description, and where such reference is lacking a rationale for their selection should be provided. It should also include the steps taken to adhere to the detection criteria.

### GROESBE Item 7a

#### Methods: Outcomes—Describe the Diagnostic Criteria and Case Definition for Untreated Dental Caries

Untreated dental caries is different from caries experience. The former reflects current disease whereas the latter includes past disease (restorations and extractions) and present disease. It is untreated dental caries that needs to be measured for burden estimation. The WHO (2013), International Caries Detection and Assessment System (ICDAS Coordinating Committee 2005), and Caries Assessment Spectrum and Treatment (CAST) criteria (Leal et al. 2017) are commonly used for detection of carious lesions, which include different disease detection thresholds. Authors are encouraged to report untreated dental caries 1) at the cavity threshold, which is preferred for burden estimation (WHO codes 1 and 2, ICDAS codes 4 to 6, and CAST codes 4 to 7) and, if available, 2) at the enamel threshold (ICDAS codes 1 to 6, CAST codes 3 to 7), which will help to understand preventive and restorative needs.

### GROESBE Item 7b

#### Methods: Outcomes—Describe the Diagnostic Criteria and Case Definition for Severe Periodontitis

There is no universally accepted case definition of severe periodontitis. To allow estimation of disease burden, we recommend adhering to the reporting standards developed by Holtfreter et al. (2015). They include thresholds ≥4 and ≥6 mm for probing pocket depth and ≥3 and ≥5 mm for clinical attachment level. Severe periodontitis could then be defined as having ≥1 periodontal sites with probing pocket depth ≥6 mm (or clinical attachment level ≥5 mm), which is the case definition used in the GBD study (Bernabe et al. 2020). For studies using the case definition of the Centers for Disease Control and Prevention/American Academy of Periodontology (AAP) (Eke et al. 2012) or the European Federation of Periodontology/AAP (Tonetti et al. 2018), we recommend additionally reporting prevalence of probing pocket depth ≥6 mm (or clinical attachment level ≥5 mm) to allow burden estimation.

### GROESBE Item 7c

#### Methods: Outcomes—Describe the Diagnostic Criteria and Case Definition for Edentulism

Complete edentulism is defined as having no natural teeth remaining, including third molars, which can be determined from clinical examination or self-assessment. According to this case definition, individuals with only retained roots, treated or not, are considered dentate. This will reduce potential disagreements between clinical and self-reported assessments of edentulism. In addition, retained and unerupted teeth are excluded when classifying individuals as dentate or edentulous because their presence cannot be confirmed during clinical examination or self-assessment (i.e., a radiographic assessment would be needed, which is not conventionally available in epidemiologic surveys).

### GROESBE Item 8

#### Methods: Data Sources/Measurement—Give Sources of Data and Details of Methods of Assessment (Examination Protocol, Reliability Assessment, and Summary Indices) for Each Oral Condition

Authors should state the sources of data (e.g., self-reported vs. clinical examination) and how data were collected for each oral condition. The examination protocol should be given, including details on 1) the method of inspection (e.g., visual, visual-tactile); 2) whether a full- or partial-mouth inspection was carried out and the areas of the mouth that were included if partial-mouth inspection was indeed used; 3) the level at which inspection and recording were carried out (person, quadrant, tooth, surface, site, etc.); 4) whether third molars were included in the examination; 5) the characteristics of the setting where examinations were performed (e.g., house, schools, mobile clinic); and 6) any equipment (e.g., dental chairs, compressed air) and instruments used (e.g., mouth mirror, periodontal probe type, light source).

The reliability of outcome assessments should be stated, including the number of examiners, their training, and the number of those assessed. Because the reliability assessment of examiners during training is not applicable to the data collected in the main study, the reliability assessment should be conducted during the main study. Reliability should be based on duplicate examinations by the same examiner and against a standard examiner (intra- and interexaminer reliability, respectively) by using the entire detection criteria, not just the presence or absence of the outcome. Authors should be clear about the type of reliability measure reported. We recommend using the kappa statistics for categorical criteria and the intraclass correlation coefficient for continuous measurements. Details on the type of kappa (i.e., simple vs. weighted and the type of weights used in the calculation) (Gwet 2021a) and intraclass correlation coefficient should be included (i.e., model: 1-way random effects, 2-way random effects, or 2-way mixed effects; type of relationship: consistency or absolute agreement; single rater/measurement or mean of multiple raters/measurements) (Gwet 2021b).

### GROESBE Item 10

#### Methods: Study Size—Explain How the Sample Size Was Determined

Authors should provide details of how the sample size was arrived at. For new studies, a priori estimates of sample size need to be reported as well as any assumptions made in the sample calculation. If data already available are used (secondary data analysis), authors need to clarify and report whether data analysis will produce results with sufficient statistical precision, especially for subgroups, as indicated by the confidence intervals.

### GROESBE Item 12a

#### Methods: Statistical Methods—Describe How Prevalence and Incidence Were Estimated

Authors should describe the approach used to estimate prevalence and incidence, including details of the specific time points at which estimates were derived. For prevalence studies, we recommend using the case definitions for untreated caries, severe periodontitis, and edentulism proposed in this guideline (see items 7a to 7c). We recommend using the total population (dentate and edentate participants) as the denominator to calculate the prevalence of untreated dental caries, severe periodontitis, and edentulism. Any adjustment to the prevalence of severe periodontitis for the bias due to the use of partial-mouth examination, such as inflation factors (Susin et al. 2005) or multiple imputation (Preisser et al. 2024), should be explained in detail and both estimates reported (before and after adjustment).

We recommend that incidence be reported by the case definitions proposed in this guideline (see items 7a to 7c). Incidence at the person level can then be expressed as the proportion of participants developing the oral condition (cumulative incidence) or as a rate per person-time of follow-up (incidence rate). We recommend using the number of dentate participants (population at risk) as the denominator to calculate the incidence of untreated dental caries, severe periodontitis, and edentulism.

When complex sampling strategies are used to recruit participants, the sampling design parameters (e.g., oversampling, clustering, stratification, and weighting) must be incorporated into the analysis to produce estimates that are representative of the source population. Authors should state whether and, if so, which sampling design parameters were included during the analysis. Measures of precision, such as standard error or confidence interval, should be corrected per the design effect: a ratio measure that describes how much precision is gained or lost if a more complex sampling strategy is used instead of simple random sampling.

### GROESBE Item 12c

#### Methods: Statistical Methods—Clearly Describe How Missing Data Were Handled

Reasons for missing values should be given when possible, indicating the number of individuals excluded because of missing data. For prevalence studies, authors should also describe any methods used to handle data missingness (e.g., nonresponse adjustment to survey weights). For incidence studies, authors are recommended to report reasons for any losses to follow-up. If more complex methods to handle missing data are used (e.g., inverse probability weighting or multiple imputation), we recommend reporting details following the Treatment and Reporting of Missing Data in Observational Studies (TARMOS) framework (Lee et al. 2021).

### GROESBE Item 13

#### Results: Participants—Report the Number of Participants at Each Stage of the Study and Reasons for Nonparticipation at Each Stage

Authors should give details of how many individuals, including percentages, were considered at each stage of the recruitment, from the target population to those included in data analysis. For prevalence studies, this may include the number of individuals who were potentially eligible, assessed for eligibility, confirmed as eligible, included in the study, examined, and included in the analysis (e.g., final sample size). For incidence studies, this will also include the number of individuals followed-up and included in the incidence analysis. Reasons for noneligibility, nonparticipation, and lack of response at each stage should be given. Some participants might become edentulous between assessments and subsequently excluded from the calculation of incidence. Therefore, authors are recommended to report whether and, if so, how many participants became edentulous for incidence studies on untreated caries and severe periodontitis. Authors might consider presenting this information in a detailed flowchart.

### GROESBE Item 14

#### Results: Descriptive Data—For Incidence Studies, Report Details of Follow-up Time

Authors should report the mean or median follow-up time (or both) and the spread of follow-up times. Reporting the mean allows the reader to calculate the total number of person-years by multiplying it with the number of study participants. The spread of follow-up times can be presented by the minimum and maximum times or percentiles of the distribution. If there were multiple assessments over time, the authors should clearly state for which period incidence estimates are reported.

### GROESBE Item 15

#### Results: Outcome Data—Report the Number of Existing and/or New Cases of the Condition

Authors should first report the number of participants with edentulism (prevalence studies) or the number of participants who became edentulous in the specified period (incidence studies). This step is not necessary when reporting surveys carried out among children. Thereafter, authors should report the number of dentate participants with untreated dental caries and severe periodontitis or the number of dentate participants who developed these conditions.

Prevalence and incidence estimates should be reported for the full sample and disaggregated by age, sex, geography (rural/urban, regions, etc.), and socioeconomic groups. Disaggregation by age will vary by the source population (preschool children, schoolchildren, adults, older adults, etc.) and available sample size. For studies among children, we recommend disaggregating prevalence and incidence estimates for those aged <5 y (primary teeth), 6 to 8 y (first stage of mixed dentition), 9 to 11 y (second stage of mixed dentition), and 12 to 14 y (permanent teeth) to account for the replacement of the dentition. From them on, we recommend disaggregating estimates in 10-y intervals (i.e., 15 to 24, 25 to 34, etc., plus a category for ≥95 y) (Diaz et al. 2021). We recommend that the numbers in each age group be reported to allow readers to ascertain the reliability of estimates.

## Discussion

GROESBE was developed to improve the reporting of descriptive oral epidemiologic studies to inform burden estimation. It is envisioned that GROESBE will help improve the quality and completeness of prevalence and incidence studies of common oral conditions and reduce the number of poorly reported studies. Furthermore, it will facilitate the inclusion of more studies in future iterations of the GBD study, thus improving quantification of disease burden nationally and globally.

GROESBE is an extension of the STROBE recommendations and, as such, should be used alongside existing guidelines. We anticipate that GROESBE will be relevant to epidemiologists, public health practitioners, global health specialists, academics, and editors with an interest in common oral conditions. We recommend that authors consult the guidelines regularly during the design and planning of new epidemiologic studies. We invite dental and public health journals to fully endorse the use of these guidelines. This could be done by listing GROESBE among the journal’s recommendations for preparation of manuscripts for submission. That said, we emphasize that GROESBE should not be used to appraise the methodological quality of oral epidemiologic studies because of the critical distinction between methodological and reporting quality, which should be reflected in the choice of quality assessment tools and reporting guidelines (da Costa et al. 2011; Faggion 2023). We also understand that reporting on all GROESBE items can make manuscripts somewhat longer, thus exceeding journals’ word limits. However, this could be addressed by presenting information in a supplemental file.

As with any other reporting guidelines, the main limitation of the GROESBE guidelines is to ensure implementation in practice (Johansen and Thomsen 2016; Pussegoda et al. 2017). This could be addressed by disseminating the guidelines widely, including the translation to other languages. The moderate participation rate in the Delphi panel is another limitation. However, current standards for Delphi panels put more emphasis on panel size and composition than on participation rate (Spranger et al. 2022). In that sense, we achieved our goal of recruiting a homogenous group that was balanced in terms of gender and geographic distribution, as it is generally recommended.

Next steps are the development of the explanation and elaboration of this guideline, which will contain further details and examples of robust reporting to facilitate the use of the guidelines. We welcome feedback and suggestions for improvement from the global oral health community as these guidelines will be updated periodically. During the next years, GROESBE will be translated. As is recommended (Moher et al. 2010), it will be important to evaluate the impact of implementation of GROESBE on reporting in future incidence and prevalence studies of common oral conditions. This could be done by comparing the proportion of prevalence and incidence studies on oral conditions that are excluded from the GBD study database before and after the publication of the guidelines.

## Conclusion

GROESBE extends the existing STROBE guidelines, providing guidance for the reporting of descriptive oral epidemiologic studies of common oral conditions. We hope that GROESBE, including 14 specific recommendations and an accompanying checklist, will facilitate reliable comparison of emerging prevalence and incidence data on common oral conditions across settings worldwide and the synthesis of robust evidence to inform estimation of disease burden in future iterations of the GBD study.

## Author Contributions

E. Bernabé, contributed to conception, design, data acquisition, analysis, and interpretation, drafted and critically revised the manuscript; C.C. Salomon-Ibarra, contributed to data acquisition and analysis, drafted the manuscript; W. Marcenes, contributed to conception, design, and interpretation, critically revised the manuscript. All authors gave final approval and agree to be accountable for all aspects of the work.

## Supplemental Material

sj-docx-1-jdr-10.1177_00220345241293410 – Supplemental material for Guidelines for Reporting Oral Epidemiologic Studies to Inform Burden Estimation (GROESBE) Supplemental material, sj-docx-1-jdr-10.1177_00220345241293410 for Guidelines for Reporting Oral Epidemiologic Studies to Inform Burden Estimation (GROESBE) by E. Bernabé, C.C. Salomon-Ibarra and W. Marcenes in Journal of Dental Research
