# Supplementary material for: Guidelines for Reporting Oral Epidemiologic Studies to Inform Burden Estimation (GROESBE)
Source: J Dent Res. 2024 Dec 5;104(2):140–6. doi: 10.1177/00220345241293410 (PMC11752649; doi:10.1177/00220345241293410)
Supplement: sj-docx-1-jdr-10.1177_00220345241293410 – Supplemental material for Guidelines for Reporting Oral Epidemiologic Studies to Inform Burden Estimation (GROESBE) [file sj-docx-1-jdr-10.1177_00220345241293410.docx]

**Appendices for Guidelines for Reporting Oral Epidemiologic Studies to inform Burden Estimation (GROESBE)**

**Authors and affiliations:**

Bernabé E^1^, Salomon-Ibarra CC^1^, Marcenes W^2^

^1^ Institute of Dentistry, Queen Mary University of London, London, United Kingdom

^2^ Affordable Health Initiative, United Kingdom

**Table of contents:**

| **Item** | **Page** |
| --- | --- |
| **Appendix 1.** Methodology and results of the GROESBE study | 2 |
| **Appendix 2.** Explanation and elaboration of GROESBE items | 5 |
| **Appendix 3.** Editable version of the GROESBE checklist | 20 |
| **Appendix 4.** Editable version of the GROESBE flowchart of participants | 12 |

**Appendix 1. Methodology and results of the GROESBE study**

**METHODS**

The present study was approved by the Ethics Research Committee of Queen Mary University of London (reference: QMERC23.155). The proposal was registered prospectively in the Enhancing the QUAlity and Transparency Of health Research (EQUATOR) Library in September 2023. The guidelines were developed in two phases following current recommendations (Moher et al. 2010).

**Phase I: Preparatory work**

The GBD systematic reviews on the epidemiology of untreated dental caries (Kassebaum et al. 2015), severe periodontitis (Kassebaum et al. 2014a) and edentulism (Kassebaum et al. 2014b) were used as a starting point to identify common limitations in published studies. This information was complemented by electronic searches of MEDLINE via Pubmed, Embase via Ovid and the EQUATOR library to identify relevant standards for reporting prevalence and incidence studies, quality assessment tools and core outcome sets. The search strategy was based on the following concepts: (1) prevalence, incidence and risk, (2) checklist reporting, guideline reporting, standard reporting and quality reporting, (3) critical appraisal, quality appraisal, quality rating, study quality, study evaluation, study assessment, quality evaluation, quality assessment and risk of bias, and (3) core outcomes.

Further eligible reports were identified through Google search and hand-searching bibliography lists of included reports. The search was conducted in December 2023. Results from the different searches were imported into Rayyan web application (Ouzzani et al. 2016), where duplicates were removed and the title and abstract of documents were screened for eligibility. The search and initial screening were completed by one reviewer (CCSI) and verified by another (EB). The full text of all relevant reports was retrieved and read by two members of the working group (CCSI and EB). This work provided normative justification to generate a list of proposed recommendations, which addressed limitations found in previous descriptive oral epidemiological studies and were not covered in existing guidelines.

**Phase II: Delphi study**

A Delphi panel was conformed to agree on the recommendations to be included in GROESBE. The working group carried out internet searches to identify and recruit potential panellists from academic and non-academic backgrounds, who had experience in either (a) conducting or analysing oral health surveys, or (b) using survey findings for research and/or policy, which was corroborated with publications indexed in Pubmed and/or personal websites. The goal was to recruit a diverse group of panellists according to gender and geographical location (i.e. from each of the six WHO regions).

Following international recommendations (Jünger et al. 2017), the Delphi study was conducted through a mix of online surveys and group discussions. Potential panellists were identified and sent an email invitation. Those who expressed interest were subsequently emailed the participant Information sheet and the link to the online survey round 1. All panellists provided consent before starting the survey. Participation was anonymous to enable panellists to comment freely and openly. Panellists were asked three questions to allow linking responses between the two surveys. In round 1, panellists were asked to score the proposed items on their relevance for inclusion in GROESBE, using a scale from 1 (strongly disagree) to 9 (strongly agree). If an item was considered relevant but poorly phrased, panellists were asked to suggest changes to the wording via open-ended boxes embedded in the survey. Items were revised to address the comments of the panellists. In round 2, items were presented in original and revised forms along with their corresponding summary scores from round 1. Panellists were asked to re-score the items and suggest any further modifications. Both surveys were developed and hosted in Qualtrics XM online platform (Qualtrics, Provo, UT, USA) and completed between February and April 2024. The online survey was piloted before round 1 to check face validity and ensure correct functionality. Scores were summarised quantitatively (response rate, mean, standard deviation, median, and interquartile range for each item) and open-ended responses summarised qualitatively (unearthing key themes from free-text information). Consensus was achieved when at least 51% of the panellists agree with the item inclusion. Otherwise, the item was omitted from the final checklist.

Two online group discussions were held in May 2024 to agree on the final list of recommendations to be included in GROESBE. A revised list of items was circulated before the meetings and panellists had the opportunity to share their views on the revised list of items during the online meeting. Discussions were informed by the scores received during the two rounds of online surveys, focusing on items that needed further clarification or improvement.

**RESULTS**

Of the 6,662 citations retrieved (after removing duplicates), 6,626 reports were excluded after screening titles and abstracts as clearly irrelevant. The full text of 36 reports was retrieved to check eligibility and 21 reports were subsequently removed as irrelevant. Therefore, a total of 15 reports were found relevant, including 6 reporting guidelines, 7 quality assessment tools and 2 systematic reviews of quality assessment tools. Of the 6 reporting guidelines identified, 1 was developed for reporting prevalence of gingival recession (Yadav et al. 2023) and 1 for reporting prevalence and severity of periodontitis (Holtfreter et al. 2015). No relevant core outcome sets were identified.

A total of 56 individuals were invited to participate in the Delphi panel. Of them, 27 (48%) expressed an interest and 23 (41%) consented to participate. The panel was geographically diverse, representing 26 countries across the 6 WHO regions (Appendix 1 Table 1). In addition, 14 panellists were female (52%), 23 were from academic institutions (85%), and 6 were from the GBD collaborators network (22%).

**Appendix 1 Table 1.** Geographical distribution of GROESBE panellists.

| **Regions** | **Number of panellists** | **Number of countries** | **List of countries** |
| --- | --- | --- | --- |
| AFR | 5 | 5 | Kenya, Nigeria, South Africa, Tanzania, Uganda |
| AMR | 4 | 4 | Chile, Brazil, El Salvador, USA |
| EMR | 5 | 5 | Egypt, Iran, Lybia, Saudi Arabia, UAE |
| EUR | 4 | 4 | Germany, Greece, Switzerland, UK |
| SEAR | 5 | 5 | India, Indonesia, Myanmar, Sri Lanka, Thailand |
| WPR | 4 | 3 | Australia, Malaysia, South Korea |
| Total | **27** | **26** |  |

Appendix 1 Table 2 summarises the responses for each GROESBE item in each round as well as the difference between rounds. Generally, there was a high level of agreement. All items had 67% or more of panellists given one of the two highest possible scores (8 or 9).

**Appendix 1 Table 2.** Scores for GROESBE items in each round of Delphi study (n=23 panellists)

| **GROESBE Item** | **Round 1** | | | **Round 2** | | |
| --- | --- | --- | --- | --- | --- | --- |
|  | **P25, P50, P75** | **Lowest, Highest** | **% with score>8** | **P25, P50, P75** | **Lowest, Highest** | **% with score>8** |
| **4.** Study design | 9, 9, 9 | 7, 9 | 95.7 | 9, 9, 9 | 7, 9 | 91.3 |
| **5.** Setting | 9, 9, 9 | 8, 9 | 100.0 | 8,9,9 | 6, 9 | 91.3 |
| **6.** Participants | 9, 9, 9 | 7, 9 | 90.9 | 8,9,9 | 7, 9 | 95.7 |
| **7.** Outcomes | 9, 9, 9 | 6, 9 | 91.3 | 8,9,9 | 5, 9 | 91.3 |
| **7a**. Untreated caries | 8, 9, 9 | 1, 9 | 82.6 | 7,8,9 | 2, 9 | 69.6 |
| **7b.** Severe periodontitis | 8, 8.5, 9 | 1, 9 | 77.3 | 8,8.5,9 | 2, 9 | 86.4 |
| **7c.** Edentulism | 8, 9, 9 | 6, 9 | 82.6 | 8,9,9 | 3, 9 | 77.3 |
| **8.** Data measurement | 8, 9, 9 | 6, 9 | 90.9 | 8,9,9 | 7, 9 | 90.5 |
| **10.** Study size | 9, 9, 9 | 5, 9 | 82.6 | 8,8,9 | 5, 9 | 81.0 |
| **12a.** Statistical methods | 8, 9, 9 | 6, 9 | 82.6 | 7,8,9 | 3, 9 | 66.7 |
| **12b.** Statistical methods | 8, 9, 9 | 5, 9 | 82.6 | 8,8,9 | 6, 9 | 90.0 |
| **13.** Participants | 8, 9, 9 | 6, 9 | 86.4 | 8,9,9 | 5, 9 | 85.7 |
| **14.** Descriptive data | 8, 9, 9 | 5, 9 | 86.4 | 8,9,9 | 7,9 | 85.7 |
| **15.** Outcome data | 8, 9, 9 | 7, 9 | 95.7 | 8,8,9 | 1,9 | 81.0 |

**REFERENCES**

Holtfreter B, Albandar JM, Dietrich T, Dye BA, Eaton KA, Eke PI, Papapanou PN, Kocher T, Joint EUUSAPEWG. 2015. Standards for reporting chronic periodontitis prevalence and severity in epidemiologic studies: Proposed standards from the joint eu/USA periodontal epidemiology working group. J Clin Periodontol. 42(5):407-412.

Jünger S, Payne SA, Brine J, Radbruch L, Brearley SG. 2017. Guidance on conducting and reporting delphi studies (credes) in palliative care: Recommendations based on a methodological systematic review. Palliat Med. 31(8):684-706.

Kassebaum NJ, Bernabe E, Dahiya M, Bhandari B, Murray CJ, Marcenes W. 2014a. Global burden of severe periodontitis in 1990-2010: A systematic review and meta-regression. J Dent Res. 93(11):1045-1053.

Kassebaum NJ, Bernabe E, Dahiya M, Bhandari B, Murray CJ, Marcenes W. 2014b. Global burden of severe tooth loss: A systematic review and meta-analysis. J Dent Res. 93(7 Suppl):20S-28S.

Kassebaum NJ, Bernabe E, Dahiya M, Bhandari B, Murray CJ, Marcenes W. 2015. Global burden of untreated caries: A systematic review and metaregression. J Dent Res. 94(5):650-658.

Moher D, Schulz KF, Simera I, Altman DG. 2010. Guidance for developers of health research reporting guidelines. PLoS Med. 7(2):e1000217.

Ouzzani M, Hammady H, Fedorowicz Z, Elmagarmid A. 2016. Rayyan-a web and mobile app for systematic reviews. Syst Rev. 5(1):210.

Yadav VS, Monga N, Jose NK, Priya H. 2023. Evidence-based suggestions to improve the methodological issues in reporting of prevalence studies on gingival recession. J Indian Soc Periodontol. 27(5):461-464.

**Appendix 2. Explanation and elaboration of the GROESBE items**

GROESBE was developed as an extension to the STROBE statement and follows the same structure. When no specific GROESBE item is listed, this means that the original STROBE item alone suffices. The GROESBE checklist provides additional details for 10 of the 22 items on the STROBE checklist. This appendix should be read in conjunction with the main manuscript and other appendices.

Our intention is to explain how to report descriptive oral epidemiological studies well, not how they should be done. Each GROESBE item is presented with an explanation and examples of what we considered clear and transparent reporting. Although examples were taken from published studies, their selection does not mean that these studies were uniformly well reported or well conducted. It simply means that this specific aspect of the methodology or results was well reported in the study.

Although we quote examples as originally reported, in some cases we had to edit the text for clarity. Some examples were edited by removing citations or spelling out abbreviations. In other cases, we present the edited text between parenthesis, either containing an ellipsis to indicate the omission of words or containing words to improve the fluency of the text. Finally, we recommend addressing all GROESBE items somewhere in the paper, but we do not prescribe a precise location or order. We give authors the freedom to address several items within a single section of text or in a table.

**THE GROESBE ITEMS**

**GROESBE item 4. METHODS: Study design - Provide a full description of the study design**

Authors should report the study design so that it is clear whether data were collected at one point in time (cross-sectional study reporting prevalence), multiple points in time for different participants (repeated cross-sectional study reporting prevalence trends) or multiple points in time for the same participants (cohort study reporting incidence).

**Examples:**

“A population-based cross-sectional study design was employed to determine the burden of dental caries in primary teeth” [1].

“This prospective cohort study conducted in Piracicaba, São Paulo, Brazil (...)” [2]

“Four cross-sectional epidemiological studies in 1973, 1983, 1993, and 2003 were performed in Jönköping, Sweden” [3].

**GROESBE item 5. METHODS: Setting - Describe the setting, locations and dates of data collection.**

Authors should provide information on the setting (e.g. recruitment sites such as communities, schools, primary care practices, hospitals, care homes) and location (e.g. geographical area) where the study took place to assess the context and generalisability of the results. Authors should also give an indication of the geographical coverage of the study, reporting whether it was local (i.e. single community), regional or national. Any claims regarding representativeness of the study sample should be explained and well founded. Authors should also state the year(s) when data collection took place.

**Examples:**

“The Tromstannen–Oral Health in Northern Norway (TOHNN) study is a population-based, cross-sectional representative study with a target population of adults aged 20 to 79 years, living in Troms County, Norway. Troms County is one of three Norwegian counties located north of the Arctic Circle. Tromsø, one of the largest cities within the Arctic Circle, surrounded by islands, fjords, and mountain peaks, and the gateway to the Polar Seas, is included in the catchment area. In January 2013, 112,253 people in the selected age group inhabited the county. (...) To obtain a representative selection of all regions in the county, the sample was stratiﬁed on three different areas: Tromsø (51,110 people: 46%), Southern Troms County (49,740 people: 44%), and northern Troms County (11,403 people: 10%). Three thousand individuals were selected by simple random sampling technique from the population register by Statistics Norway, resulting in 1,380 people from Tromsø, 1,320 people from Southern Troms County, and 300 people from Northern Troms County.” [4]

“A cross-sectional oral-health national survey of 12-year-old children was conducted in 2016. (...) According to 2011 statistics, Latvia has 2,070,371 inhabitants with a wide ethnic diversity including Latvian (61.8%), Russian (25.6%), Belarusian (3.4%), Ukrainian (2.3%), Polish (2.1%), Lithuanian (1.2%) and others (3.6%) (...). The population is distributed across 110 municipalities and 9 large cities, with the greatest concentration of people being found in and around the port and capital city of Riga; small agglomerations are scattered throughout the rest of the country. The urban population is 68.1% of the total population. (...) The approximate population of 12-year-olds was 11,028 in 2016. (...) Only 12-year-olds were included. Participants were recruited from 92 out of 487 schools that had courses for 12-year-olds” [5].

“The present study corresponds to a cohort with 7 years of follow-up in the city of Santa Maria, southern Brazil. The first stage (T1) took place in 2010, consisting of a systematic sample of 639 children aged 1 to 5 years. In 2010, the city had a population of approximately 261,031 citizens, among them, 27,520 were children under 6 years old. The sampling process included municipal health centers on the National Children’s Vaccination Day. The selected health centers were those with a dental chair (15 of 28 health centers), being distributed in different neighborhoods and covering all the 8 administrative regions of the city. Also, about 85% of children vaccinated that day were seen by the selected health centers” [6].

**GROESBE item 6. METHODS: Participants - Describe the source population, eligibility criteria and methods of selection of participants. For incidence studies, describe methods of follow-up.**

A description of the population from which participants were selected (i.e., general population, non-institutionalised population, schoolchildren, working-age adults) and any eligibility criteria (i.e. inclusion and exclusion criteria) should be presented. Eligibility criteria should be justified as they could limit the representativeness of the sample (Locker 2000).

Both the method of recruitment (e.g. email/postal invitation, existing records, referrals, self-selection through advertisements) and sampling procedure should be reported. Any methods used to increase participation and retention of participants should also be reported. It must be stated whether a probabilistic (e.g. participants recruited using simple random, systematic, cluster or stratified sampling or a combination of those as in multi-stage sample) or non-probabilistic sample (e.g. convenience, WHO pathfinder survey) was used.

For incidence studies, authors should state the length of follow-up. It is also recommended to give details on how participants were recontacted (e.g. electronic methods, non-electronic methods, record linkage) and whether similar procedures were used for all participants.

Examples:

“The target population comprised adults, aged between 20 and 75 years, living in Turin (Italy). (...) 1600 individuals were randomly selected from the Health Regional Register of Piedmont using a stratiﬁed two-stage sampling design. The Health Regional Register collects demographic information of the entire population resident in Turin grouped according to the state-provided general practitioners (GPs) to whom they are assigned. In Italy all residents are covered by the National Health System, assigned a public GP and enrolled in the regional health registries. The primary sampling units were GPs stratiﬁed by the four districts of Turin to ensure geographic and socioeconomic coverage over the whole of Turin. The probability of being selected was proportional to the number of subjects attending each GP. The second stage units were the subjects cared for by each GP, who were sampled using a random sampling technique. Overall, 20 GPs were sampled, and 1600 patients were selected and invited to participate in the study through an invitation letter, explaining the purpose of the study and including a thorough description of the clinical examination” [7].

“The Icelandic Oral Health Survey is a national survey of dental health and related factors of children in 1st, 7th and 10th school-grade. (...) School classes of 1st, 7th and 10th grade corresponding to ages 6, 12 and 15 years were selected but schools with nine or fewer students per grade in all age-groups were excluded. Schools were sorted by size and divided into four quartiles. Subsequently, schools from each size-quartile and each place of residence, eight strata in all, were randomly selected until the desired number of participants was obtained. (...) Requests for participation were sent to school headmasters, and out of 37 requests, 31 acceptances were received. (...) The 31 schools that accepted to participate contained 20.1% of the country population in the target age groups and this comprised the original study sample (...) Parents or guardians of the children received information on the study and informed-consent forms, delivered to the children by their teachers. Nonrespondents were contacted 2 weeks later by phone and encouraged to participate” [8].

“This paper reports results from a prospective cohort study of a random sample of non-institutionalized people aged 60+ years living in two South Australian cities; Adelaide, the state capital with a population of 1.1 million; and Mt Gambier, a regional city in the south-east of the state with a population of 24000. The cohort consisted of dentate people who were examined at baseline in 1991/92 and followed up 2 years later. (...) At baseline, a stratified random sample of people aged 60+ years was selected from the South Australian Electoral Commission's database, which is a compulsory register for Australian citizens. In Adelaide there were 18 strata identified by three age groups, two sexes and three locality categories, and in Mt Gambier there were six strata defined by three age groups and two sexes. Subjects were excluded if they lived in nursing homes or hospitals but included if they lived in hostels for the aged. (...) Dentate people who had an oral examination at baseline were recontacted approximately 2 years later, through an initial follow-up letter that was sent to their household. Then, they were called by telephone and a trained interviewer sought their participation in the follow-up part of the study” [9].

**GROESBE item 7a. METHODS: Outcomes - Describe the diagnostic criteria and case definition for untreated dental caries.**

Untreated dental caries is different from caries experience. The former reflects current disease whereas the latter includes both past (restorations and extractions) and present disease. It is untreated dental caries that needs to be measured for burden estimation. The WHO (2013), International Caries Detection and Assessment System (ICDAS Coordinating Committee 2005) and the Caries Assessment Spectrum and Treatment (CAST) criteria (Leal et al. 2017) are commonly used for detection of carious lesions, which include different disease detection thresholds. Authors are encouraged to report untreated dental caries (i) at the cavity threshold which is preferred for burden estimation (WHO codes 1-2, ICDAS codes 4-6, and CAST codes 4-7) and, if available, (ii) at the enamel threshold (ICDAS code 1-6, CAST codes 3-7), which will help to understand both preventive and restorative needs.

Examples:

“The criteria recommended by the WHO (2013) were followed for the diagnosis of dental caries. (...) A tooth was recorded as decayed when a lesion had an unmistakable cavity, undermined enamel, or detectably softened floor or wall. If a child had at least one decayed tooth, it was considered a case of caries” [10].

“The caries status was assessed according to the ICDAS-II criteria (ICDAS Coordinating Committee 2005). (...) To allow comparability of the results obtained using the ICDAS-II criteria with studies using the WHO criteria, the cut-off point was established in code 4 of ICDAS-II. Following from this, ICDAS-II values 4, 5, and 6 comply with the WHO criteria cavity definition. (...) The variables calculated for the study were caries prevalence (ICDAS-II 2-6>0 and ICDAS-II 4-6>0)” [11].

“The dental caries in the permanent dentition was examined according to the WHO (1997) criteria. Dental caries was detected at the caries into the dentine threshold. The prevalence of untreated dental caries was defined as the existence of untreated dental caries (yes versus no)” [12].

**GROESBE item 7b. METHODS: Outcomes - Describe the diagnostic criteria and case definition for severe periodontitis.**

There is no universally accepted case definition of severe periodontitis. To allow estimation of disease burden, we recommend adhering to the reporting standards developed by Holtfreter et al. (2015). They include thresholds of ≥4 and ≥6mm for probing pocket depth (PPD) and ≥3 and ≥5mm for clinical attachment level (CAL). Severe periodontitis could then be defined as having one or more periodontal sites with PPD≥6mm (or CAL≥5mm), which is the case definition used in the Global Burden of Disease (GBD) study (Bernabe et al. 2020). For studies using the case definition of the Centers for Disease Control and Prevention/American Academy of Periodontology (CDC/AAP) (Eke et al. 2012) or the European Federation of Periodontology/AAP (EFP/AAP) (Tonetti et al. 2018), we recommend additionally reporting prevalence of PPD≥6mm (or CAL≥5mm) to allow burden estimation.

Examples:

“The periodontal status of each participant was evaluated using the Community Periodontal Index (CPI) developed by the WHO (Ainamo et al. 1982). (...) The periodontal status of 10 index teeth, 2 molars in each posterior sextant, and the upper right and lower left central incisors, were measured to evaluate whether there was periodontitis in each sextant (WHO, 1997). (...) The CPI score for each sextant was recorded in 5 levels (0 to 4) and a score of 3 or 4 indicated a section with periodontitis. For this study, we considered everyone with periodontitis in any section of the sextant(s) as a participant with periodontitis” [13].

“Prevalence of periodontitis cases was described according to (...) the case definition for the surveillance of periodontitis described by the AAP-CDC (Page et al., 2012). (...) According to this system severe periodontitis cases are identified as having≥2 interproximal sites with CAL≥6mm, and ≥1 site with PD≥5mm. (...) Prevalence of periodontitis was (also) calculated measuring the presence of at least one site with CAL≥3mm or ≥5mm, and PD≥4mm or ≥6mm (Holtfreter et al., 2015)” [14].

“The clinical attachment loss (CAL) for each site was computed by summing gingival recession and pocket depth in the unit-record data sets of periodontal data from the baseline and 5-year examinations. After the data sets were merged, the 5-year incidence of periodontal attachment loss (ALOSS) for each site was computed by subtracting baseline CAL from the follow-up CAL. (…) Data on the person-level incidence (…) are presented in Table 2 using three case definition thresholds: (i) 1+ sites with ALOSS of 3+mm, (ii) 1+ sites with ALOSS of 4+mm and (iii) 1+ sites with ALOSS of 5+mm” [15].

**GROESBE item 7c. METHODS: Outcomes - Describe the diagnostic criteria and case definition for edentulism.**

Complete edentulism is defined as having no natural teeth remaining (including third molars), which can be determined from clinical examination or self-assessment. According to this case definition, individuals with only retained roots (treated or not) are considered dentate. This will reduce potential disagreements between clinical and self-reported assessments of edentulism. In addition, retained and unerupted teeth are excluded when classifying individuals as dentate or edentulous because their presence cannot be confirmed during clinical examination or self-assessment (i.e. a radiographic assessment would be needed, which is not conventionally available in epidemiological surveys).

Examples

“Edentulism was defined as a person who had none of the natural teeth including third molars, calculated based on the result of the oral examination” [16].

“The study outcome, edentulism, was defined as absence of natural teeth. Participants were asked about the number of missing teeth and classified as edentulous if they reported they had lost all natural teeth and as dentate if they had lost fewer than 32 teeth” [17].

“Participants were examined at baseline and follow-up, with tooth loss recorded when an intact tooth or retained root recorded as present at baseline was absent at follow-up. Incidence of edentulism was defined as the absence of teeth at follow-up” [18].

**GROESBE item 8. METHODS: Data sources/measurement - Give sources of data and details of methods of assessment (examination protocol, reliability assessment and summary indices) for each oral condition.**

Authors should state the sources of data (e.g. self-reported versus clinical examination) and how data were collected for each oral condition. The examination protocol should be given, including details on: (i) the method of inspection (e.g. visual, visual-tactile); (ii) whether a full- or partial-mouth inspection was carried out and the areas of the mouth that were included if partial-mouth inspection was indeed used; (iii) the level at which inspection and recording were carried out (i.e., person, quadrant, tooth, surface, site, etc.); (iv) whether third molars were included in the examination; (v) the characteristics of the setting where examinations were performed (e.g. house, schools, mobile clinic); and vi) any equipment (e.g. dental chairs, compressed air) and instruments used (e.g. mouth mirror, periodontal probe type, light source).

The reliability of outcome assessments should be stated, including the number of examiners, their training and the number of those assessed. Because the reliability assessment of examiners during training is not applicable to the data collected in the main study, the reliability assessment should be conducted during the main study. Reliability should be based on duplicate examinations by the same examiner and against a standard examiner (intra- and inter-examiner reliability respectively) using the entire detection criteria not just the presence or absence of the outcome. Authors should be clear about the type of reliability measure reported. We recommend using the Kappa statistics for categorical criteria and the intra-class correlation coefficient (ICC) for continuous measurements. Details on the type of Kappa (i.e. simple versus weighted and the type of weights used in the calculation) (Gwet 2021a) and ICC should be included (i.e. model: one-way random effects, two-way random effects, or two-way mixed effects; type of relationship: consistency or absolute agreement; single rater/measurement or mean of multiple raters/measurements) (Gwet 2021b).

Examples:

“The clinical oral examinations were conducted by ﬁve ﬁeld teams, each including a dentist and a dental nurse. Examinations were conducted with a portable unit, headlamp as the light source, and using a dental mirror, a WHO periodontal probe, a ﬁbre optic light, and a letter scale. Teeth identiﬁcation and clinical examination were performed according to the recommendations of the WHO (1997). A reference dentist took the parallel measurements (n=269) on several visits to each ﬁeld team. All teeth surfaces were examined, but observations were recorded by tooth. The presence of teeth included all teeth and tooth remnants visible and tactile in the mouth. The teeth were blown dry, and the status of each tooth surface was diagnosed and combined into one recording per tooth. The overall Kappa value for inter-examiner reliability, where field examiners were compared with the reference examiner in parallel measurements on 269 subjects under field circumstances, was 0.87 (95% CI: 0.84–0.89) at tooth level. The overall Kappa value for intra-examiner reliability on 111 subjects was 0.95 (95% CI: 0.871.04) at tooth level” [19].

“The clinical examinations were performed in a mobile examination unit consisting of a trailer equipped with a complete dental unit, comprising a dental chair, light, compressor, and other basic amenities. The unit was moved from one location to the next according to the survey schedule. Four periodontists assisted by two dental assistants performed the clinical examinations. (...) Trained dental assistants recorded the data on prepared record sheets. All permanent fully erupted teeth, excluding third molars, were examined with a manual periodontal probe (PCP10-SE, Hu-Friedy Inc., Chicago, IL) color-coded at 1, 2, 3, 5, 7, 8, 9, and 10 mm. Six sites per tooth were assessed in the mesio-buccal, mid-buccal, disto-buccal, disto-lingual, mid-lingual, and mesio-lingual sites. Probing depth was deﬁned as the distance from the free gingival margin to the bottom of the pocket/sulcus. Gingival recession was deﬁned as the distance from the cemento-enamel junction (CEJ) to the free gingival margin and was assigned a negative sign if the gingival margin was located coronal to the CEJ. Clinical attachment loss (CAL) was deﬁned as the distance from the CEJ to the bottom of the pocket/sulcus and was calculated as the sum of the probing depth and gingival recession measurements. Measurements were made in millimeters, rounded to the lower whole millimeter. (...) At two time points, before and 3 months after the start of the study, the examiners were trained and calibrated in performing the clinical measurements. The examination team followed a quality control protocol aimed at reducing systematic and random measurement errors and to quantify what error remained. The protocol involved standard examination environment and methodology, standard equipment, and detailed written instructions for clinical procedures. Assessment of measurement reproducibility used replicate periodontal measurements performed during the ﬁeldwork. The examiner with the most clinical experience served as the gold standard examiner. A total of 57 subjects, divided into four groups ranging from eight to 20 subjects, were used for the reproducibility assessment. In one of the groups, the replicate measurements consisted of repeated measurements by the gold standard examiner. In the remaining three groups, the replicate measurements were made by one examiner and the gold standard examiner. Measurement reproducibility at the subject level was assessed by the intra-class correlation coefﬁcient and weighted kappa, and at the site level by the weighted Kappa. The intraclass correlation coefﬁcient for mean CAL ranged between 0.95 and 0.99 and for extent scores of CAL ≥5mm and ≥7mm ranged between 0.80 and 0.98. The weighted kappa (±1 mm) at subject level prevalence (maximum attachment loss) were between 0.69 and 1.00. The weighted kappa (±1 mm) at site level ranged between 0.65 and 0.87” [20].

“The nurses were trained by nine dentists associated with the Ministry of Health of Chile (MINSAL). Also, MINSAL provided the nurses with a handbook for conducting dental assessments called “The Nurse Manual.” This handbook is available in the national repository of population-based surveys (http://epi.minsal.cl/encuestas-poblacionales/). According to the pilot study of the ENS 2003, the nurses’ sensitivity to detect tooth loss was over 70%, and the inter-examiner reliability was significant (kappa 0.75, p-value<0.001). (…) The nurses wore gloves, surgical masks (and used) head flashlights, tongue depressors and flat mouth mirrors. Clinical examination was carried out while the participant was sitting in a straight back chair in front of the light from the head flashlight. To determine the number of remaining teeth, a nurse asked (participants) to brush their teeth previously and remove their prosthesis; then, they counted the participants’ teeth in each dental arch” [21].

**GROESBE item 10. METHODS: Study size - Explain how the sample size was determined.**

Authors should provide details of how the sample size was arrived at. For new studies, a priori estimates of sample size need to be reported as well as any assumptions made in the sample calculation. If data already available are used (secondary data analysis), authors need to clarify and report whether data analysis will produce results with sufficient statistical precision (especially for subgroups) as indicated by the confidence intervals.

Examples:

“The minimum required sample size was determined using standard methods to address the specific aims of the Second Australian National Child Oral Health Survey (NCOHS-2). The main outcomes evaluated were (...) and the percentage of children with untreated decay (...). These outcomes were evaluated for primary dentition, among the age groups of 5-6, 7-8, and 9-10 years, and permanent dentition, among the age groups of 6-8, 9-11, and 12-14 years. The sample size was calculated to detect (...) a 40% difference in the prevalence of the outcome with a power of 80% (between any two territories) and a significance level of 5%. Sample size requirements were calculated for a simple random sample and then adjusted by the design effect to account for the NCOHS-2 stratified, two-stage sampling design. The design effect values were estimated based on existing NCOHS 2012-2014 data, which included information on children’s oral health status (for all abovementioned outcomes) and were used as a proxy in the calculation formula to estimate sample sizes for the NCOHS-2. (...) The minimally required overall sample size of the NCOHS-2 was based on the outcome that required the largest sample –the percentage of children with untreated decay in the permanent dentition (n=16,678)– meaning that the NCOHS-2 would be adequately powered to detect all other outcomes” [22].

“Geographically, the country is divided into five strata comprising Bangkok and four regions (north, south, central and northeast). The four regions are divided into 12 health sectors. One health sector consists of two provinces and one province consists of four districts, except for Bangkok. For Bangkok, six sub-districts were randomly selected. (…) The sample size within each selected area was based on the municipal and rural population proportion, calculated using the dental caries prevalence in each age group obtained from the 7th Thai National Oral Health Survey, a relative δ (margin of error) of 10-15%, a 95% confidence interval and a design effect of two” [23].

“To calculate the representative sample of adults (20-64 years) living in Piracicaba, São Paulo, oral health conditions were assessed in different age groups and two different calculations were estimated for the sample size of young adults (20-44 years) and older adults (45-64 years). We adopted a design effect of (…1.7); margin of error of 10.0%; and 95.0% confidence interval, data concerning the prevalence of caries for each age group (70.2% and 90.9%, respectively), and added (…25%) to the total to compensate (…for potential non-response). The sample size for adults aged 20-44 years was 172, and for those aged 45-64 years, 68, totalling 240 adults. We added 30% to the final sample size for selecting adults, foreseeing the possibility of losses and refusals (to follow-up), resulting in (…312) households” [24].

**GROESBE item 12a. METHODS: Statistical methods - Describe how prevalence and incidence were estimated.**

Authors should describe the approach used to estimate prevalence and incidence, including details of the specific time point(s) at which estimates were derived. For prevalence studies, we recommend using the case definitions for untreated caries, severe periodontitis and edentulism proposed in this guideline (see items 4a-4c). We recommend using the total population (dentate and edentate participants) as the denominator to calculate the prevalence of untreated dental caries, severe periodontitis and edentulism.

We also recommend that incidence be reported using the case definitions proposed in this guideline (see items 4a-4c). Incidence at person-level can then be expressed as the proportion of participants developing the oral condition (cumulative incidence) or as a rate per person-time of follow-up (incidence rate). We recommend using the number of dentate participants (population at risk) as the denominator to calculate incidence of untreated dental caries, severe periodontitis and edentulism.

When complex sampling strategies are used to recruit participants, the sampling design parameters (e.g., oversampling, clustering, stratification, and weighting) must be incorporated into the analysis to produce estimates that are representative of the source population. Authors should state whether, and if so which, sampling design parameters were included during the analysis. Measures of precision such as standard error or confidence interval should be corrected using the design effect, a ratio measure that describes how much precision is gained or lost if a more complex sampling strategy is used instead of simple random sampling.

Examples:

“The prevalence of untreated dental caries was defined by the appearance of at least one deciduous tooth with this condition, that is, with the d component of the dmft index equal to or greater than one. (…) Descriptive analysis was carried out for (…) the prevalence of untreated dental caries (d≥1) for each state capital and for the towns in the interior of each macro-region. (…) Analyses were carried out considering the complex cluster sample design and the respective sampling weight” [25].

“For analysis, we classiﬁed participants with respect to periodontal disease using two deﬁnitions: National Health and Nutrition Examination Survey (NHANES) and the Centers for Disease Control/American Academy of Periodontology (CDC/AAP) deﬁnitions. (…) We used both deﬁnitions to allow for comparisons among studies using these deﬁnitions and because the deﬁnitions capture different aspects and extents of periodontal disease. For both the NHANES and CDC/AAP deﬁnitions, edentulism was deﬁned as not having any natural teeth remaining. All statistical analyses were survey weighted (…)” [26].

“Cumulative incidence represented the proportion of children who developed new caries within the total observation period. This proportion was calculated by counting the number of children whose caries increment exceeded zero during the observation period and then dividing by the total number of children” [27].

**METHODS: GROESBE item 12c. Statistical methods - Clearly describe how missing data was handled.**

Reasons for missing values should be given when possible, indicating the number of individuals excluded because of missing data. For prevalence studies, authors should also describe any methods used to handle data missingness (e.g., non-response adjustment to survey weights). For incidence studies, authors are recommended to report reasons for any losses to follow-up. If more complex methods to handle missing data (e.g., inverse probability weighting or multiple imputation) are used, we recommend reporting details following the Treatment And Reporting of Missing data in Observational Studies (TARMOS) framework (Lee et al. 2021).

Examples

Of the 1196 (30%) who consented to participate and completed the face-to-face interviewer-administered questionnaire, 663 (55%) participants went on to complete the clinical examination with 105 excluded from the periodontal examination for various medical reasons. (…) The study sample was weighted to account for (i) unequal probability of selection during sampling (base weights), (ii) non-response among selected households and (iii) non-response among selected individuals for the interview and clinical examination” [28].

“1021 dyads of a child and a caregiver completed data collection in wave 1. The combined screening and interviewing response rate in wave 1 was 74%. (…) Participants were followed in wave 2, when 77% (n=790) returned to complete an interview and clinical examination. (…) All analyses were adjusted with the weight developed to account for unequal selection probabilities and differential non-response” [29].

“Response rates in 2018/2019 were 53.8% (Edmonton) and 46.7% (Calgary) for schools, and 44.5% (Edmonton) and 43.7% (Calgary) for students in participating schools (i.e. those with both oral examination and questionnaire data). We developed sampling weights that accounted for the probability of selection (as per the sampling frame) and the probability of non-response, thus increasing the extent to which our samples resembled the underlying target populations” [30].

**GROESBE item 13. RESULTS: Participants - Report the number of participants at each stage of the study, and reasons for non-participation at each stage.**

Authors should give details of how many individuals (including percentages) were considered at each stage of the recruitment, from the target population to those included in data analysis. For prevalence studies, this may include the number of individuals who were potentially eligible, assessed for eligibility, confirmed as eligible, included in the study, examined and included in the analysis (e.g. final sample size). For incidence studies, this will also include the number of individuals followed-up and included in the incidence analysis. Reasons for non-eligibility, non-participation, and lack of response at each stage should be given. Some participants might become edentulous between assessments and subsequently excluded from the calculation of incidence. Therefore, authors are recommended to report whether, and if so, how many, participants became edentulous for incidence studies on untreated caries and severe periodontitis. Authors might consider presenting this information in a detailed flowchart.

Examples:

“All 152 upper-tier local authorities (LAs) took part in the survey resulting in estimates being available for 324 out of 326 lower-tier Las (in England). Consent return levels varied between and within regions. From the randomly drawn samples an average of 63.1% of children were examined. Non-response to the request was the most common reason for non-consent (28.9%), despite two requests and schools actively seeking returned forms. A small proportion of parents (4.5%) returned forms stating they did not want their children included in the survey, while 0.5% of children with consent declined to take part on the day. Absences on the day of examination accounted for a loss of 3.8% of consented children. Of the children with parental consent 111,500 had valid home postcodes and so were included in the final analysis, representing 95.7% of the main consented sample. This represented 16.5% of the population of this age cohort attending mainstream state schools” [31].

“After exclusion of migrated (n=615) and deceased persons (n=126), the net sample included 6265 eligible subjects of whom 4308 subjects participated, which corresponds to a response of 68.8%. For 4288 of 4308 subjects with an oral examination, 515 subjects were edentulous in the examined side. In 37 subjects, periodontal examinations were either refused or not recordable because of medical reasons, resulting in 3736 subjects with probing depth data. Further, attachment loss could not be determined in 185 subjects mainly due to crowns resulting in 3551 subjects with available attachment (loss) values” [32].

“(At baseline), all 12-year-olds, 4355 in total, were invited by letter to a screening session for caries (…) and 3373 agreed to take part in the study and were examined for caries. Of the 3373 12-year-olds examined in 1995, 3109 were again examined in 1997. Full examinations for both years were performed on 3107 teenagers and it is these (individuals who) provided the (incidence) data. Of the 266 teenagers who were not examined on both occasions, 231 formally left the study, and the rest did not show up for the second examination. Of the 231 who formally left the study, 122 had moved from the area, 74 did not want to, or could not, participate any longer, 24 said they wanted to change their dentist, and 11 were taken out of the study because they had developed such caries that the treating dentist wanted to be free to use any preventive measure” [33].

**Examples of participation flowcharts:**

**
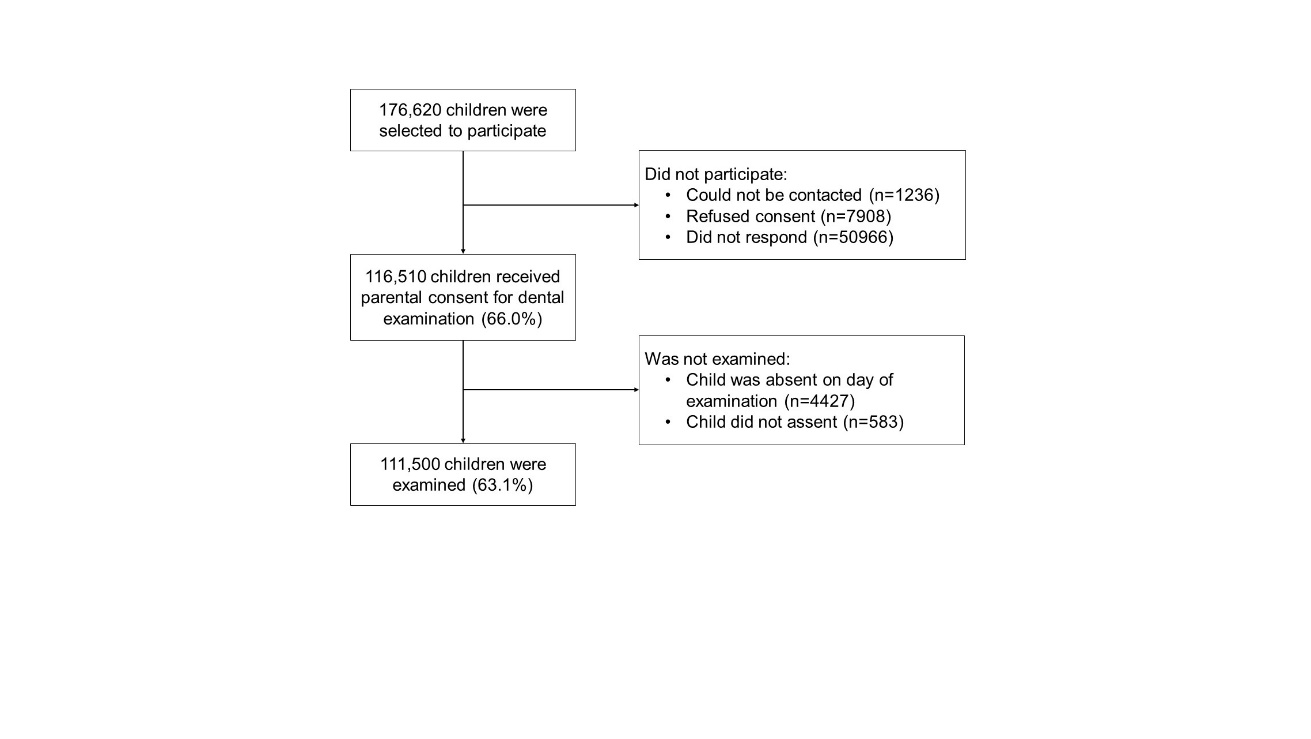
**

**Figure.** Participant flowchart, Oral Health Survey of 5-year-old children (2014-15) in England. Based on data presented by Jones et al. [31]

**
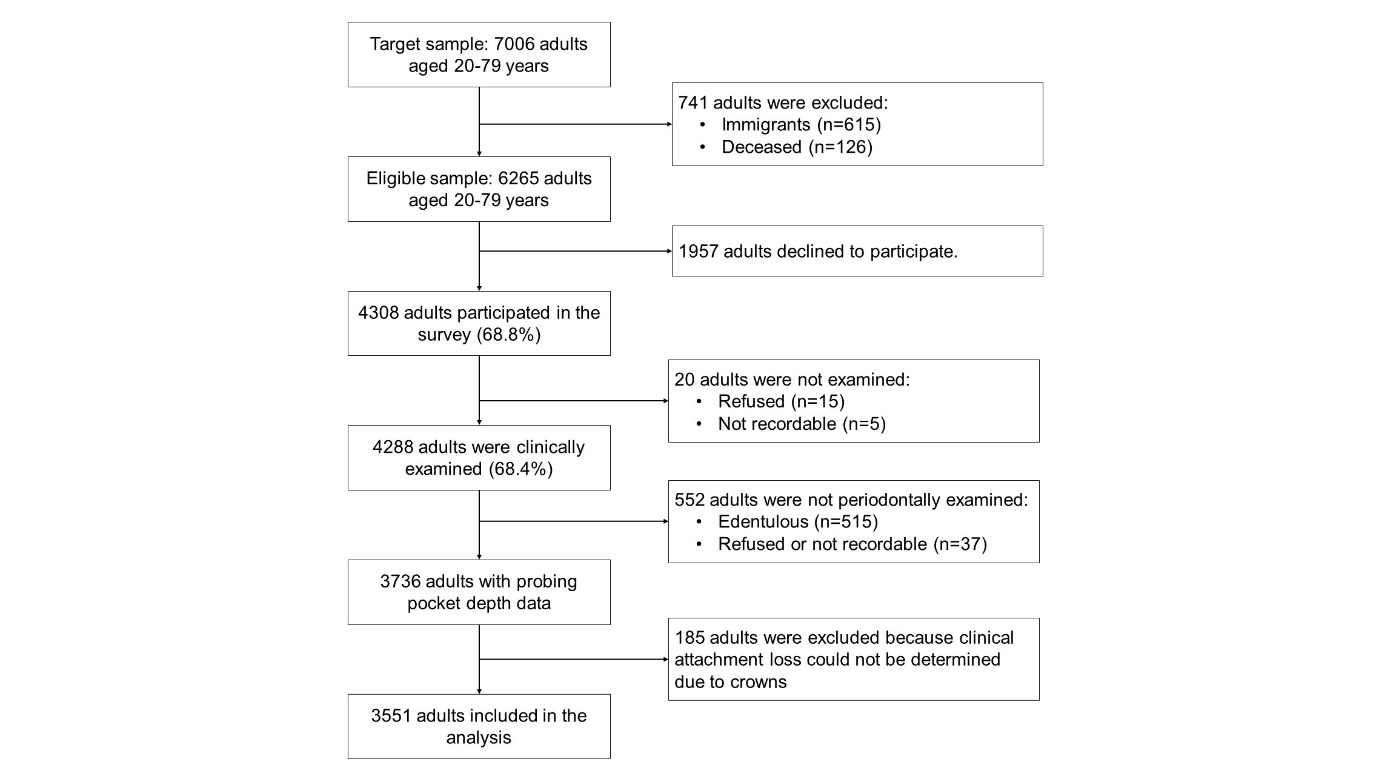
**

**Figure.** Flow of participants through the Study of Health in Pomerania (SHIP-0). Based on information presented in Schutzhold et al. [32].

**
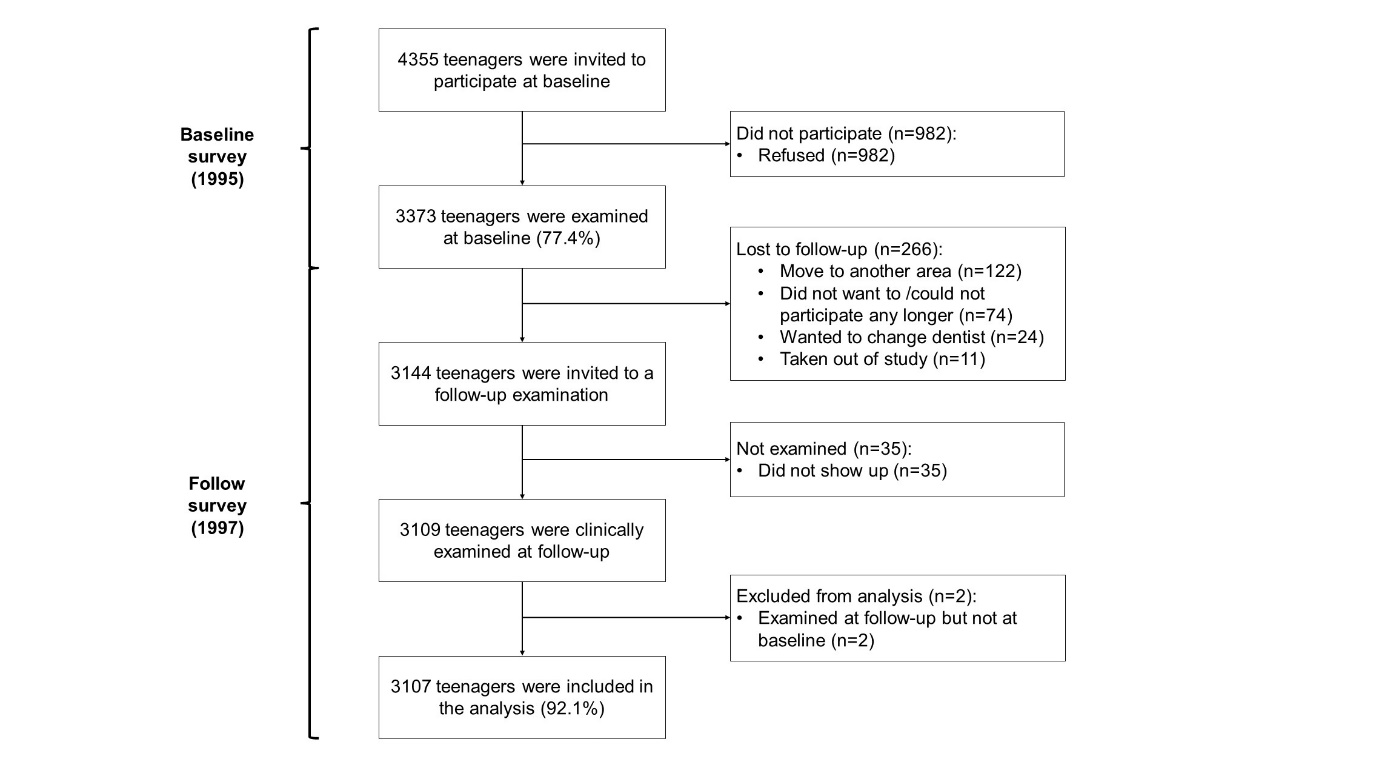
**

**Figure.** Flowchart for participants in the baseline and follow-up studies. Based on data reported by Källestål et al. [33].

**GROESBE item 14. RESULTS: Descriptive data - For incidence studies, report details of follow-up time.**

Authors should report the mean or median follow-up time (or both) and the spread of follow-up times. Reporting the mean allows the reader to calculate the total number of person-years by multiplying it with the number of study participants. The spread of follow-up times can be presented using the minimum and maximum times or percentiles of the distribution. If there were multiple assessments over time, the authors should clearly state for which period incidence estimates are reported.

Examples:

“The mean time between baseline and follow-up examinations was 13.9 years, with a range of 13-15 years” [34].

Follow-up time varied between 4.4 and 8.5 years, mean 5.26 (95% conﬁdence interval: 5.24-5.28)” [35].

“The mean follow-up time from baseline and first follow-up was 5.1 ± 0.4 years (P25=5.0, P50 =0.50 and P75: 5.1 years) in the Study of Health in Pomerania (SHIP)-START and 7.38 ± 0.65 (P25=7.04, P50=7.28 and P75=7.50 years) in SHIP-TREND” [36].

**GROESBE item 15. RESULTS: Outcome data - Report the number of existing and/or new cases of the condition.**

Authors should first report the number of participants with edentulism (prevalence studies) or the number of participants who became edentulous in the specified period (incidence studies). This step is not necessary when reporting surveys carried out among children. Thereafter, authors should report the number of dentate participants with untreated dental caries and severe periodontitis or dentate participants who developed these conditions.

Prevalence and incidence estimates should be reported for the full sample and disaggregated by age, sex, geography (i.e. rural/urban, regions, etc.) and socioeconomic groups. Disaggregation by age will vary depending on the source population (e.g., pre-school children, schoolchildren, adults, older adults, etc.) and available sample size. For studies among children, we recommend disaggregating prevalence and incidence estimates for <5 (primary teeth), 6-8 (first stage of mixed dentition), 9-11 (second stage of mixed dentition) and 12-14-year-olds (permanent teeth) to account for the replacement of the dentition. From them on, we recommend disaggregating estimates in 10-year intervals (i.e. 15-24, 25-34, and so on, plus a category for 95 years or older) (Diaz et al. 2021). We recommend that the numbers in each age group be reported to allow readers ascertain the reliability of estimates.

Examples

“The sample consisted of 1197 15-19-year-old adolescents (participation rate: 72.3%), being 513 males (42.9%) and 684 females (57.1%). (…) The prevalence of untreated caries was 26% (n=312)” [37].

“Among the 1,983 participants, ≥65 years of age, in the National Health and Nutrition Examination Survey (NHANES) 2009-2012, fewer than one (19%) in five was edentulous: one-seventh (13.7%) of the 65-74-year-old subjects were edentulous compared with one-quarter (24.1%) of those ≥75 years of age. Periodontal and all covariable data were complete for 1,511 dentate individuals (…). Almost two-thirds (62.3%) of adults had one or more sites with clinical attachment loss of ≥5 mm. (…) Almost half of the older adults (48.3%) had at least one site with periodontal probing depth≥4mm and over 11.9% had one or more sites with periodontal probing depth≥6 mm” [38].

“During the 60 months between baseline and the final follow-up, 106 subjects had died, 25 were too ill to continue participation, 10 had moved from the area, and 44 refused to participate. Five-year follow-up examinations were completed on 340 of the 525 dentate subjects examined at baseline. (...) Five persons (1.5% of the sample) had lost all their remaining teeth (...) Six people not examined at the 5-year follow-up, but who were examined at one of the interim exams (at 1.5, 3 and 4 years), also became edentulous. Table 2 shows (...) the percentage of persons who (became edentulous over the follow-up period) by age group and sex” [39].

**Examples of tables reporting prevalence/incidence by subgroups:**

**Appendix 2 Table 1.** Prevalence (%) of dental caries in the primary and permanent dentition for children and adolescents in the United States 2011-2014. Adapted from Dye et al. [40].

| **Dentition** | **Age group** | **Poverty status** | **Total** | | **Males** | | **Females** | |
| --- | --- | --- | --- | --- | --- | --- | --- | --- |
|  |  |  | **%** | **SE** | **%** | **SE** | **%** | **SE** |
| Primary | 2-5 years | Poor | 17.6 | 2.1 | 16.6 | 2.6 | 18.6 | 2.2 |
|  |  | Borderline | 11.0 | 1.6 | 9.9 | 2.1 | 11.4 | 2.5 |
|  |  | Non-poor | 6.2 | 1.0 | 7.6 | 2.0 | 5.3 | 1.5 |
|  |  | Total | 11.0 | 1.0 | 10.9 | 1.3 | 11.1 | 1.4 |
|  | 6-8 years | Poor | 22.3 | 2.8 | 25.7 | 3.4 | 18.6 | 3.7 |
|  |  | Borderline | 22.1 | 3.4 | 24.7 | 5.6 | 19.5 | 3.2 |
|  |  | Non-poor | 11.7 | 1.4 | 11.5 | 2.3 | 10.9 | 2.3 |
|  |  | Total | 17.4 | 1.7 | 18.7 | 2.3 | 15.8 | 1.7 |
|  | 2-8 years | Poor | 19.6 | 1.9 | 20.5 | 2.2 | 18.6 | 2.2 |
|  |  | Borderline | 15.7 | 1.9 | 16.2 | 2.8 | 14.8 | 2.2 |
|  |  | Non-poor | 8.6 | 0.9 | 9.2 | 1.2 | 7.7 | 1.4 |
|  |  | Total | 13.7 | 1.1 | 14.2 | 1.4 | 13.1 | 1.1 |
| Permanent | 6-8 years | Poor | 5.6 | 1.2 | 4.3 | 1.4* | 6.7 | 1.9 |
|  |  | Borderline | 3.7 | 1.1 | DSU | DSU | DSU | DSU |
|  |  | Non-poor | 1.9 | 0.6* | DSU | DSU | DSU | DSU |
|  |  | Total | 3.4 | 0.6 | 2.9 | 0.6 | 3.9 | 1.0 |
|  | 9-11 years | Poor | 11.3 | 1.5 | 11.0 | 2.4 | 11.5 | 2.9 |
|  |  | Borderline | 8.8 | 2.5 | 6.1 | 2.3* | 11.3 | 3.3 |
|  |  | Non-poor | 6.5 | 1.4 | 7.9 | 2.4* | 4.8 | 1.3 |
|  |  | Total | 8.4 | 1.0 | 8.5 | 1.4 | 8.1 | 1.1 |
|  | 6-11 years | Poor | 8.4 | 0.9 | 7.7 | 1.2 | 9.1 | 1.6 |
|  |  | Borderline | 6.3 | 1.6 | 4.7 | 1.4 | 8.0 | 2.4 |
|  |  | Non-poor | 4.3 | 0.8 | 5.0 | 1.3 | 3.2 | 0.8 |
|  |  | Total | 5.9 | 0.6 | 5.7 | 0.7 | 6.0 | 0.8 |
|  | 12-15 years | Poor | 25.0 | 3.3 | 24.8 | 3.4 | 25.1 | 5.1 |
|  |  | Borderline | 18.2 | 2.4 | 23.2 | 4.4 | 11.8 | [2.9](https://www.sciencedirect.com/science/article/pii/S0002817717303483#tbl4fnhash) |
|  |  | Non-poor | 8.1 | 1.4 | 9.0 | 1.9 | 7.2 | [2.4*](https://www.sciencedirect.com/science/article/pii/S0002817717303483#tbl4fndaggerdagger) |
|  |  | Total | 14.5 | 1.2 | 16.4 | 1.5 | 12.7 | [1.8](https://www.sciencedirect.com/science/article/pii/S0002817717303483#tbl4fnhash) |
|  | 16-19 years | Poor | 26.7 | 2.9 | 31.6 | 4.5 | 22.9 | 2.8 |
|  |  | Borderline | 26.7 | 2.9 | 30.5 | 5.3 | 23.0 | 4.8 |
|  |  | Non-poor | 16.3 | 3.1 | 15.4 | 3.0 | 17.1 | 4.1 |
|  |  | Total | 22.5 | 1.9 | 23.6 | 2.5 | 21.3 | 2.8 |
|  | 12-19 years | Poor | 25.9 | 2.0 | 28.3 | 3.1 | 23.9 | 2.7 |
|  |  | Borderline | 22.6 | 2.2 | 27.0 | 4.0 | 17.6 | 3.4 |
|  |  | Non-poor | 12.3 | 1.8 | 12.3 | 1.8 | 12.3 | 2.7 |
|  |  | Total | 18.6 | 1.3 | 20.1 | 1.6 | 17.1 | 1.9 |

DSU: Data statistically unreliable (relative standard error>40%). SE: Standard Error.

* Estimate should be interpreted with caution (relative standard error>30% but ≤40%).

**Appendix 2 Table 2:** Prevalence of untreated caries (DT≥1) according to participants’ sociodemographic and socioeconomic status (n=451). Adapted from Idon et al. [41]

|  | | **Total, n (%)** | | **Prevalence** | |
| --- | --- | --- | --- | --- | --- |
|  | | **n** | **(%)** | **n** | **(%)** |
| Age group | |  |  |  |  |
|  | <25 years | 146 | (32.4) | 130 | (89.0) |
|  | 25-34 years | 135 | (29.9) | 111 | (82.2) |
|  | 35-44 years | 110 | (24.4) | 88 | (80.0) |
|  | 45-54 years | 39 | (8.6) | 27 | (69.2) |
|  | 55-64 years | 14 | (3.1) | 9 | (64.3)* |
|  | 65-74 years | 7 | (1.6) | 3 | (42.9)* |
| Gender | |  |  |  |  |
|  | Male | 257 | (57.0) | 202 | (78.6) |
|  | Female | 194 | (43.0) | 166 | (85.6) |
| Education level | |  |  |  |  |
|  | No formal education | 33 | (7.3) | 30 | (90.9) |
|  | Below tertiary education | 121 | (26.8) | 108 | (89.3) |
|  | Tertiary education | 297 | (65.9) | 230 | (77.4) |
| Residence | |  |  |  |  |
|  | Rural | 435 | (96.5) | 355 | (81.6) |
|  | Urban | 16 | (3.5) | 13 | (81.3)* |
| Total | | 451 | (100) | 368 | (81.6) |

* Estimates based on fewer than 30 people are considered unstable.

**Appendix 2 Table 3.** Prevalence of edentulism by sex and age groups (n=1553). Adapted from Urzia et al. [42]

|  | **35-44 years** | | **65-74 years** | |
| --- | --- | --- | --- | --- |
| **Gender** | **n**^a^ | **% (95% CI)** | **n**^a^ | **% (95% CI)** |
| Female | 597 | 0.33 (−0.13, 0.80) | 288 | 13.89 (9.88, 17.9-) |
| Male | 491 | 0.20 (0.19, 0.60) | 177 | 7.34 (3.48, 11.21) |
| All | 1088 | 0.28 (−0.03, 0.58) | 465 | 11.4 (8.49, 14.29) |

^a^ Unweighted number of participants examined.

**REFERENCES**

1. Alhabdan YA, Albeshr AG, Yenugadhati N, Jradi H: Prevalence of dental caries and associated factors among primary school children: a population-based cross-sectional study in Riyadh, Saudi Arabia. Environ Health Prev Med 2018, 23(1):60.

2. Silva-Junior MF, Batista MJ, de Sousa M: Incidence of Tooth Loss in Adults: A 4-Year Population-Based Prospective Cohort Study. Int J Dent 2017, 2017:6074703.

3. Hugoson A, Sjödin B, Norderyd O: Trends over 30 years, 1973-2003, in the prevalence and severity of periodontal disease. J Clin Periodontol 2008, 35(5):405-414.

4. Holde GE, Oscarson N, Trovik TA, Tillberg A, Jonsson B: Periodontitis Prevalence and Severity in Adults: A Cross-Sectional Study in Norwegian Circumpolar Communities. J Periodontol 2017, 88(10):1012-1022.

5. Maldupa I, Sopule A, Uribe SE, Brinkmane A, Senakola E: Caries Prevalence and Severity for 12-Year-Old Children in Latvia. Int Dent J 2021, 71(3):214-223.

6. Costa MD, Brondani B, Knorst JK, Mendes FM, Ardenghi TM: Number of dentists in the neighborhood and incidence of dental caries in the children permanent dentition. Braz Dent J 2022, 33(4):103-112.

7. Aimetti M, Perotto S, Castiglione A, Mariani GM, Ferrarotti F, Romano F: Prevalence of periodontitis in an adult population from an urban area in North Italy: findings from a cross-sectional population-based epidemiological survey. J Clin Periodontol 2015, 42(7):622-631.

8. Agustsdottir H, Gudmundsdottir H, Eggertsson H, Jonsson SH, Gudlaugsson JO, Saemundsson SR, Eliasson ST, Arnadottir IB, Holbrook WP: Caries prevalence of permanent teeth: a national survey of children in Iceland using ICDAS. Community Dent Oral Epidemiol 2010, 38(4):299-309.

9. Slade GD, Gansky SA, Spencer AJ: Two-year incidence of tooth loss among South Australians aged 60+ years. Community Dent Oral Epidemiol 1997, 25(6):429-437.

10. Sultana S, Parvin MS, Islam MT, Chowdhury EH, Bari ASM: Prevalence of Dental Caries in Children in Mymensingh and Its Associated Risk Factors: A Cross-Sectional Study. Dent J (Basel) 2022, 10(7).

11. Almerich-Torres T, Montiel-Company JM, Bellot-Arcis C, Iranzo-Cortes JE, Ortola-Siscar JC, Almerich-Silla JM: Caries Prevalence Evolution and Risk Factors among Schoolchildren and Adolescents from Valencia (Spain): Trends 1998-2018. Int J Environ Res Public Health 2020, 17(18).

12. Lee HJ, Han DH: Exploring the determinants of secular decreases in dental caries among Korean children. Community Dent Oral Epidemiol 2015, 43(4):357-365.

13. Hwang EC, Hwang HA, Shin SY, Kim J, Kim JH: Association between dietary quality and the prevalence of periodontitis in older Korean adults aged 60 or over. J Periodontal Implant Sci 2023.

14. Serrano C, Suarez E: Prevalence of Severe Periodontitis in a Colombian Adult Population. J Int Acad Periodontol 2019, 21(2):53-62.

15. Thomson WM, Slade GD, Beck JD, Elter JR, Spencer AJ, Chalmers JM: Incidence of periodontal attachment loss over 5 years among older South Australians. J Clin Periodontol 2004, 31(2):119-125.

16. Park HA, Shin SH, Ryu JI: Edentulous disparities among geriatric population according to the sexual difference in South Korea: a nationwide population-based study. Sci Rep 2023, 13(1):7854.

17. Menegazzo GR, Cunha ARD, Fagundes MLB, Amaral Júnior OLD, Giordani J, Hilgert JB, Abreu LG, Hugo FN: Pathways that explain racial differences on edentulism among older adults: 2019 Brazil National Health Survey. Braz Oral Res 2023, 37:e40.

18. Marques FP, Tôrres LH, Bidinotto AB, Hilgert JB, Hugo FN, De Marchi RJ: Incidence and predictors of edentulism among south Brazilian older adults. Community Dent Oral Epidemiol 2017, 45(2):160-167.

19. Bernabé E, Vehkalahti MM, Sheiham A, Aromaa A, Suominen AL: Sugar-sweetened beverages and dental caries in adults: a 4-year prospective study. J Dent 2014, 42(8):952-958.

20. Susin C, Dalla Vecchia CF, Oppermann RV, Haugejorden O, Albandar JM: Periodontal attachment loss in an urban population of Brazilian adults: effect of demographic, behavioral, and environmental risk indicators. J Periodontol 2004, 75(7):1033-1041.

21. Ortuño D, Martínez C, Caneo C, Paredes F, Soto M, González MI, Vargas JP, Koller G: Tooth loss and depression in Chilean participants of the National Health Survey 2016-2017: Oral and social functions mediation analysis. J Affect Disord 2024, 358:19-27.

22. Jamieson L, Luzzi L, Chrisopoulos S, Roberts R, Arrow P, Kularatna S, Mittinty M, Haag D, Ribeiro Santiago PH, Mejia G: Oral Health, Social and Emotional Well-Being, and Economic Costs: Protocol for the Second Australian National Child Oral Health Survey. JMIR Res Protoc 2023, 12:e52233.

23. Kaewkamnerdpong I, Harirugsakul P, Prasertsom P, Vejvithee W, Niyomsilp K, Gururatana O: Oral status is associated with chewing difficulty in Thai older adults: data from a National Oral Health Survey. BMC Oral Health 2023, 23(1):35.

24. Silva-Junior MF, Batista MJ, De Sousa MDLR: Incidence of tooth loss in adults: A 4‐year population‐based prospective cohort study. Int J Dent, 2017, 6074703.

25. Ardenghi TM, Piovesan C, Antunes JL: [Inequalities in untreated dental caries prevalence in preschool children in Brazil]. Rev Saude Publica 2013, 47 Suppl 3:129-137.

26. Huang Y, Michaud DS, Lu J, Platz EA: The association of clinically determined periodontal disease and edentulism with total cancer mortality: The National Health and Nutrition Examination Survey III. Int J Cancer 2020, 147(6):1587-1596.

27. Vanobbergen J, Martens L, Lesaffre E, Bogaerts K, Declerck D: The value of a baseline caries risk assessment model in the primary dentition for the prediction of caries incidence in the permanent dentition. Caries Res 2001, 35(6):442-450.

28. Sim CPC, Lee YH, Sim YF, Wong J, Soh SH, Goh BT, Poon CY, Allen PF, Chia KS, Wong ML: Findings from the 2019 nationally representative oral health survey for adults in Singapore. Community Dent Oral Epidemiol 2024, 52(3):281-291.

29. Ismail AI, Sohn W, Lim S, Willem JM: Predictors of dental caries progression in primary teeth. J Dent Res 2009, 88(3):270-275.

30. McLaren L, Patterson SK, Faris P, Chen G, Thawer S, Figueiredo R, Weijs C, McNeil D, Waye A, Potestio M: Fluoridation cessation and children's dental caries: A 7-year follow-up evaluation of Grade 2 schoolchildren in Calgary and Edmonton, Canada. Community Dent Oral Epidemiol 2022, 50(5):391-403.

31. Jones CM, Davies GM, Monaghan N, Morgan MZ, Neville JS, Pitts NB: The caries experience of 5 year-old children in Scotland in 2013-2014, and in England and Wales in 2014-2015. Reports of cross-sectional dental surveys using BASCD criteria. Community Dent Health 2017, 34(3):157-162.

32. Schutzhold S, Kocher T, Biffar R, Hoffmann T, Schmidt CO, Micheelis W, Jordan R, Holtfreter B: Changes in prevalence of periodontitis in two German population-based studies. J Clin Periodontol 2015, 42(2):121-130.

33. Källestål C, Wall S: Socio-economic effect on caries. Incidence data among Swedish 12-14-year-olds. Community Dent Oral Epidemiol 2002, 30(2):108-114.

34. Warren JJ, Watkins CA, Cowen HJ, Hand JS, Levy SM, Kuthy RA: Tooth loss in the very old: 13-15-year incidence among elderly Iowans. Community Dent Oral Epidemiol 2002, 30(1):29-37.

35. Buchwald S, Kocher T, Biffar R, Harb A, Holtfreter B, Meisel P: Tooth loss and periodontitis by socio-economic status and inflammation in a longitudinal population-based study. J Clin Periodontol 2013, 40(3):203-211.

36. Kocher T, Meisel P, Biffar R, Völzke H, Holtfreter B: The natural history of periodontal disease-Part 2: In populations with access to dental care: The Studies of Health in Pomerania (SHIP). Periodontol 2000 2023.

37. Dalla Nora A, Knorst JK, Comim LD, Racki DNO, Alves LS, Zenkner JEA: Is neighborhood income associated with untreated dental caries irrespective of family income? Clin Oral Investig 2022, 26(7):4929-4934.

38. Eke PI, Wei L, Borgnakke WS, Thornton-Evans G, Zhang X, Lu H, McGuire LC, Genco RJ: Periodontitis prevalence in adults ≥ 65 years of age, in the USA. Periodontol 2000 2016, 72(1):76-95.

39. Hand JS, Hunt RJ, Kohout FJ: Five-year incidence of tooth loss in Iowans aged 65 and older. Community Dent Oral Epidemiol 1991, 19(1):48-51.

40. Dye BA, Mitnik GL, Iafolla TJ, Vargas CM: Trends in dental caries in children and adolescents according to poverty status in the United States from 1999 through 2004 and from 2011 through 2014. J Am Dent Assoc 2017, 148(8):550-565.e557.

41. Idon PI, Ikusika OF, Ogundare TO, Yusuf J, Enone LL, Aliyu AB: Associations of Untreated Caries and Experience among WHO-Recommended Adult Age Groups. Nigerian Journal of Medicine 2022, 31(1):59-67.

42. Urzua I, Mendoza C, Arteaga O, Rodriguez G, Cabello R, Faleiros S, Carvajal P, Munoz A, Espinoza I, Aranda W et al: Dental caries prevalence and tooth loss in Chilean adult population: first national dental examination survey. Int J Dent 2012, 2012:810170.

**Appendix 3. Editable version of the GROESBE checklist**

| **Item** | **Item number** | **STROBE Recommendations** | **GROESBE items** |
| --- | --- | --- | --- |
| **Title and abstract** | 1 | (*a*) Indicate the study’s design with a commonly used term in the title or the abstract. |  |
|  |  | (*b*) Provide in the abstract an informative and balanced summary of what was done and what was found. |  |
| **Introduction** |  |  |  |
| Background/rationale | 2 | Explain the scientific background and rationale for the investigation being reported. |  |
| Objectives | 3 | State specific objectives, including any prespecified hypotheses. |  |
| **Methods** |  |  |  |
| Study design | 4 | Present key elements of study design early in the paper. | GROESBE 4. Provide a full description of the study design. |
| Setting | 5 | Describe the setting, locations, and relevant dates,  including periods of recruitment, exposure, follow-up, and data collection. | GROESBE 5. Describe the setting, locations and dates of data collection. |
| Participants | 6 | *(a) Cohort study*—Give the eligibility criteria, and the sources and methods of selection of participants. Describe methods of follow-up.  *Case-control study*—Give the eligibility criteria, and the sources and methods of case ascertainment and control selection. Give the rationale for the choice of cases and controls.  *Cross-sectional study*—Give the eligibility criteria, and the sources and methods of selection of participants. | GROESBE 6. Describe the source population, eligibility criteria and methods of selection of participants. For incidence studies, describe methods of follow-up. |
|  |  | *(b) Cohort study*—For matched studies, give matching criteria and number of exposed and unexposed  *Case-control study*—For matched studies, give matching criteria and the number of controls per case |  |
| Variables | 7 | Clearly define all outcomes, exposures, predictors, potential confounders, and effect modifiers. Give diagnostic criteria, if applicable. | GROESBE 7. Outcomes - Describe the diagnostic criteria for each oral condition. |
|  |  |  | GROESBE 7a. Describe the diagnostic criteria and case definition for untreated dental caries. |
|  |  |  | GROESBE 7b. Describe the diagnostic criteria and case definition for severe periodontitis. |
|  |  |  | GROESBE 7c. Describe the diagnostic criteria and case definition for edentulism. |
| Data sources/ measurement | 8 | For each variable of interest, give sources of data and details of methods of assessment (measurement). Describe comparability of assessment methods if there is more than one group. | GROESBE 8. Give sources of data and details of methods of assessment (examination protocol, reliability assessment and summary indices) for each oral condition. |
| Bias | 9 | Describe any efforts to address potential sources of bias. |  |
| Study size | 10 | Explain how the study size was arrived at. | GROESBE 10. Explain how the sample size was determined. |
| Quantitative variables | 11 | Explain how quantitative variables were handled in the analyses. If applicable, describe which groupings were chosen and why. |  |
| Statistical methods | 12 | (a) Describe all statistical methods, including those used to control for confounding. | GROESBE 12a. Describe how prevalence and incidence were estimated. |
|  |  | (b) Describe any methods used to examine subgroups and interactions. |  |
|  |  | (c) Explain how missing data were addressed. | GROESBE 12c. Clearly describe how missing data was handled. |
|  |  | (d) Cohort study—If applicable, explain how loss to follow-up was addressed.  Case-control study—If applicable, explain how matching of cases and controls was addressed.  Cross-sectional study—If applicable, describe analytical methods taking account of sampling strategy. |  |
|  |  | (e) Describe any sensitivity analyses. |  |
| **Results** |  |  |  |
| Participants | 13 | (a) Report numbers of individuals at each stage of study—eg numbers potentially eligible, examined for eligibility, confirmed eligible, included in the study, completing follow-up, and analysed. | GROESBE 13. Report the number of participants at each stage of the study, and reasons for non-participation at each stage |
|  |  | (b) Give reasons for non-participation at each stage. |  |
|  |  | (c) Consider use of a flow diagram. |  |
| Descriptive data | 14 | (a) Give characteristics of study participants (eg demographic, clinical, social) and information on exposures and potential confounders. | GROESBE 14. For incidence studies, report details of follow-up time. |
|  |  | (b) Indicate number of participants with missing data for each variable of interest. |  |
|  |  | (c) *Cohort study*—Summarise follow-up time (eg, average and total amount). |  |
| Outcome data | 15 | *Cohort study*—Report numbers of outcome events or summary measures over time. | GROESBE 15. Report the number of existing and/or new cases of the condition. |
|  |  | *Case-control study*—Report numbers in each exposure category, or summary measures of exposure. |  |
|  |  | *Cross-sectional study*—Report numbers of outcome events or summary measures. |  |
| Main results | 16 | *(a)* Give unadjusted estimates and, if applicable, confounder-adjusted estimates and their precision (eg, 95% confidence interval). Make clear which confounders were adjusted for and why they were included. |  |
|  |  | *(b)* Report category boundaries when continuous variables were categorized. |  |
|  |  | *(c)* If relevant, consider translating estimates of relative risk into absolute risk for a meaningful time period. |  |
| Other analysis | 17 | Report other analyses done—e.g. analyses of subgroups and interactions, and sensitivity analyses. |  |
| **Discussion** |  |  |  |
| Key results | 18 | Summarise key results with reference to study objectives. |  |
| Limitations | 19 | Discuss limitations of the study, taking into account sources of potential bias or imprecision. Discuss both direction and magnitude of any potential bias. |  |
| Interpretation | 20 | Give a cautious overall interpretation of results considering objectives, limitations, multiplicity of analyses, results from similar studies, and other relevant evidence. |  |
| Generalisability | 21 | Discuss the generalisability (external validity) of the study results. |  |
| **Other information** |  |  |  |
| Funding | 22 | Give the source of funding and the role of the funders for the present study and, if applicable, for the original study on which the present article is based. |  |

GROESBE: Guidelines for reporting Oral Epidemiologic Studies to inform Burden Estimation; STROBE: Strengthening the reporting of observational studies in epidemiology.

**Appendix 4. Editable version of the GROESBE flowchart of participants**

Target population (n)

assessed for eligibility (n)

Participated in the study (n, %)

Included in prevalence analysis (n, %)

Included in incidence analysis (n, %)

Not assessed for eligibility (n):

- Reason A (n)
- …

Ineligible (n):

- Reason B (n)
- …

Eligible but not recruited (n):

- Reason C (n)
- …

Not examined (n):

- Reason D (n)
- …

Excluded from analysis (n):

- Reason E (n)
- …

Ineligible/excluded (n):

- Reason D (n)
- …

Lost to follow-up (n):

- Reason E (n)
- …

**Incidence studies**

**Prevalence studies**
